# Multi Source Electric Vehicles: Smooth Transition Algorithm for Transient Ripple Minimization

**DOI:** 10.3390/s22186772

**Published:** 2022-09-07

**Authors:** Adel Oubelaid, Nabil Taib, Toufik Rekioua, Mohit Bajaj, Vojtech Blazek, Lukas Prokop, Stanislav Misak, Sherif S. M. Ghoneim

**Affiliations:** 1 Laboratoire de Technologie Industrielle et de l’Information, Faculté de Technologie, Université de Bejaia, 06000 Bejaia, Algeria; 2 Department of Electrical Engineering, Graphic Era (Deemed to be University), Dehradun 248002, India; 3 ENET Centre, VSB—Technical University of Ostrava, 708 00 Ostrava, Czech Republic; 4 Department of Electrical Engineering, College of Engineering, Taif University, P.O. Box 11099, Taif 21944, Saudi Arabia

**Keywords:** hybrid electric vehicle, soft transition algorithm, transition function, operating point, fuel cell (FC), supercapacitor (SC)

## Abstract

Any engineering system involves transitions that reduce the performance of the system and lower its comfort. In the field of automotive engineering, the combination of multiple motors and multiple power sources is a trend that is being used to enhance hybrid electric vehicle (HEV) propulsion and autonomy. However, HEV riding comfort is significantly reduced because of high peaks that occur during the transition from a single power source to a multisource powering mode or from a single motor to a multiple motor traction mode. In this study, a novel model-based soft transition algorithm (STA) is used for the suppression of large transient ripples that occur during HEV drivetrain commutations and power source switches. In contrast to classical abrupt switching, the STA detects transitions, measures their rates, generates corresponding transition periods, and uses adequate transition functions to join the actual and the targeted operating points of a given HEV system variable. As a case study, the STA was applied to minimize the transition ripples that occur in a fuel cell-supercapacitor HEV. The transitions that occurred within the HEV were handled using two proposed transition functions which were: a linear-based transition function and a stair-based transition function. The simulation results show that, in addition to its ability to improve driving comfort by minimizing transient torque ripples and DC bus voltage fluctuations, the STA helps to increase the lifetime of the motor and power sources by reducing the currents drawn during the transitions. It is worth noting that the considered HEV runs on four-wheel drive when the load torque applied on it exceeds a specified torque threshold; otherwise, it operates in rear-wheel drive.

## 1. Introduction

Recently, the e-vehicle market has increasingly gained momentum because of the high comfort offered by these types of vehicles such as their ability to eliminate many tasks or at least make them easy for drivers. Since e-vehicles are ecologic and environment friendly, all countries worldwide have reoriented their policies toward electrification of their transport sectors that already suffer from greenhouse gas emissions. This has led to extensive research on topics related to the field of hybrid electric vehicles such as: the control of electric propulsion systems [1,2,3,4], energy management [5,6,7,8], power electronics [9], power train architecture [10], vehicle safety [11], intelligent vehicle control [12,13], HEV fuel optimization [14], and HEV machine design [15]. Currently, the latter research topic is among the greatest trends in the field of automotive engineering. HEV autonomy is measured by the number of kilometers that can be undergone without being charged. To increase this characteristic, researchers have thought of increasing the number of power sources on board an HEV [16], which solves the autonomy problem but does not optimize HEV space. In addition, it makes the power management of an HEV power complex and it comes at the expense of vehicle cost and weight. A common power source combination used in HEVs is a supercapacitor/fuel cell that have complementary characteristics which makes their association very efficient [17]. HEV traction can be ensured using a variety of traction machines. A permanent magnet synchronous machine (PMSM) [18] is the most distinguished type of machine that is used for vehicle traction, mainly because of its high torque to mass ratio which means that smaller-sized PMSMs can develop high torque values and save space on board the HEV. HEV security and propulsion power are also enhanced by increasing the number of traction machines. Another advantage of using multi machine vehicles is the ability of running under different driving modes such as: rear-wheel drive (RWD), front-wheel drive (FWD) and four-wheel drive (4WD).

As mentioned earlier, multiple motor, multi power, and multisource electric vehicles are better in terms of reliability, autonomy, and power propulsion. However, the comfort of these vehicles is reduced during transitions. For example, when *turning off* a fast dynamic power source such as a supercapacitor and turning on a slow dynamic power source such as a fuel cell, the vehicle will experience a lack of power and large dangerous transient currents [19]. This lack of power is due to the fact that the fuel cell needs time to reach the requested power; however, the power demand is abrupt. This phenomenon reduces vehicle comfort and may cause power source damage and significant DC bus fluctuations. In addition, when an HEV toggles from single to multiple motor traction, the newly activated motors immediately receive an abrupt high torque value and the driver feels an unsuitable and uncomfortable shake.

The authors of [20] powered an HEV using a battery and fuel cells; however, both of these power sources are known for having slow dynamics. Hence, if an important amount of power was required during a short period of time, it could not be delivered instantaneously because of the slow response of both of the power sources used. In [21], the authors applied artificial intelligence techniques to control an HEV powered by fuel cells, a battery, and a PV panel. The use of three power sources enhanced HEV autonomy, but it was at the expense of its weight, cost, and power management complexity. In addition, the implementation of intelligent techniques required advanced digital hardware and a large numerical memory to handle the computations. The authors of [22] discussed the use of an NPC seven-level inverter to increase the lifetime of FC. High FC power and voltage peaks are noticed during transitions from one power level to another and from traction to regenerative braking mode and this may damage the FC or reduce its lifetime. The authors of [23] discussed the use of DTC-based fuzzy logic for HEV control and showed its effectiveness; however, during transitions, significant torque peaks and DC bus voltage fluctuations were noticed. Furthermore, the authors used two PMSM machines to power each of the rear wheels which meant that, in case of failure of one of the traction machines or in case of synchronism loss between the right and left wheels, the HEV could skid away.

In this work, the torque and the speed of front and rear PMSMs that ensure HEV traction are controlled using the direct torque control (DTC) algorithm chosen mainly for the fast and precise torque response it provides. The power sources are controlled using a PI-based control loop that allowed them to operate at the desired power levels. Although the convenient controller design performs for traction machines and power sources, significant DC bus fluctuations, and power/current peaks are still noticed during power sources switching. In addition, high torque ripples take place during the HEV drivetrain toggling from RWD to 4WD, and vice versa. The reason for the significant peaks is that the abrupt switchings violate power source dynamics, as shown later. For instance, as soon as the SC turns on, FC extinction subjects the HEV to power excess during the entire switching period, because the SC, known for its fast dynamics, rapidly achieves its targeted power, however, the FC needs more time to be turned off. Hence, the sum of the SC and FC powers is more than what is required, and this causes significant fluctuations throughout the transient period.

The novel soft transition algorithm that is proposed in this study is simple and does not include advanced mathematical modeling, which makes its implementation easy and requires less time complexity as compared with the control technique discussed in [24,25,26]. In addition, the novel soft transition algorithm permits the control of the switching period duration and offers the designer the possibility of choosing adequate transition functions that fit the transient dynamic characteristics of the power sources and traction motors undergoing transition. The proposed STA is used along with a power management strategy to provide smooth transitions between the power sources. In this study, the HEV architecture increases HEV comfort by offering automatic switching between RWD and 4WD modes. While the torque applied on the vehicle is below a specified threshold value, only the rear PMSM is used for traction. As soon as the load torque exceeds the threshold, the STA smoothly divides the load torque over the two traction machines.

In this study, the STA was used for ripple minimization during power source switchings and during RWD/4WD drivetrain commutations. It is worth noting that this promising transition technique can also be used in any engineering discipline involving transitions. During transitions, the STA acquires the actual and the next power source operating points of a given HEV system variable, it measures the transition rate, it determines a transition period, and then joins them using a transition function. In this study, linear-based transition (LBT) and stair-based transition (SBT) are used to ensure smooth HEV transitions. A comparison is made between the two proposed transition functions which are, in turn, compared to classical abrupt switching.

## 2. Model-Based Control Strategy

### 2.1. Problem Formulation

**Figure 1:**
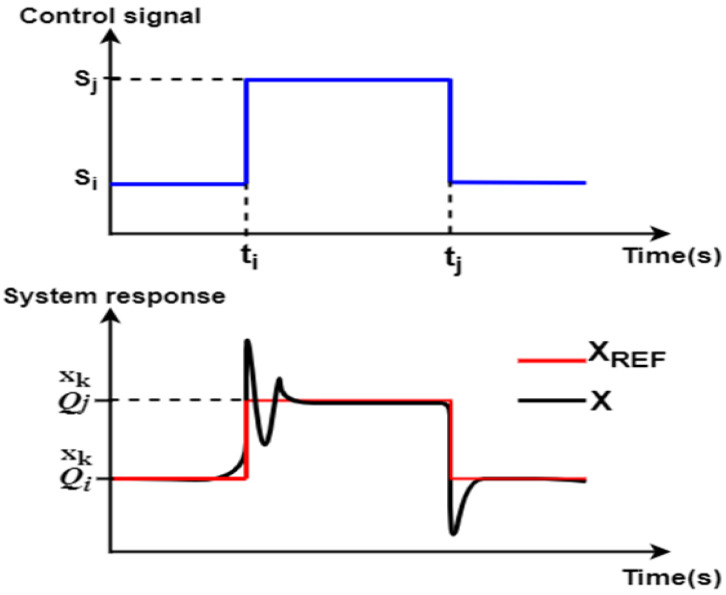
Classical abrupt switching.

When the load torque applied on a vehicle is significant and exceeds a defined threshold, more traction machines must be used. During accelerations, power sources with high power density such as a supercapacitor are needed. Hence, HEV motors and power sources are subjected to multiple on and off switching as a vehicle undergoes a driving cycle. The classical abrupt switching of power sources and traction motors is very harmful since it subjects the motor or the power source to an abrupt large current value which can damage the power source or reduce vehicle riding comfort. The classical abrupt switching mentioned earlier is a transient event that consists of changing the reference operating point “Q” of a given system variable from one value to another within zero time. Figure 1 shows one of the drawbacks of classical abrupt switching, in which high peaks and an overshoot can be observed during transitions. These peaks are not the result of bad regulation, but are mainly caused by violation of the power source dynamics, as shown in the next section.

**Figure 2:**
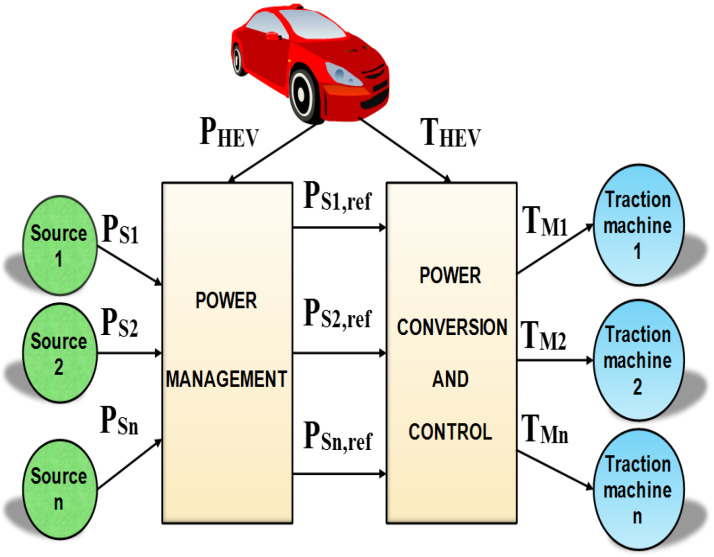
Multi power, multisource, and multiple motor HEV.

Embedded systems that are in permanent interaction with the external environment, such as HEVs, are subjected to permanent high risks of failure. One way of increasing their reliability, autonomy, and security is to reduce or eliminate vehicle dependency on one traction machine or one power source. Distributing the tasks of vehicle traction and power generation over several power sources and motors would reduce the probability of HEV failure and would enhance its performance. Figure 2 shows a general scheme of an HEV with *n* power sources and traction motors. The power sources are controlled using a power management algorithm that ensures the required type and amount of power for traction. The power delivered by the power sources is converted, controlled, and then, fed to traction motors that produce the required mechanical power for the HEV traction.

### 2.2. Control Strategy Derivation

The required traction power of an *n* source vehicle is expressed by Equation (1). It should be noted that the HEV power is the sum of the powers produced by the different power sources used:

(1) $$P_{HEV}=P_{ref}^{S_{1}}+P_{ref}^{S_{2}}+\cdots +P_{ref}^{S_{n}}$$

Equation (1) can be rewritten as follows:

(2) $$P_{HEV}=r_{s_{1}}P_{HEV}+r_{s_{2}}P_{HEV}+\cdots +r_{s_{n}}P_{HEV}$$

where *r_Si_* is a unitless factor ranging from 0 to 100% that represents the power contribution of the *i*th power source, as shown in Equation (3), where $P_{ref}^{S_{i}}$ is the power source operating point, and *P_HEV_* is the required HEV power for traction:

(3) $$r_{s_{i}}=\frac{P_{ref}^{S_{i}}}{P_{HEV}}$$

All power sources’ contribution factors sum to one, as is highlighted by Equation (4) shown below:

(4) $$\sum_{i=1}^{n}r_{s_{i}}=1$$

The torque required for HEV traction is the sum of torque produced by each traction machine, as expressed in Equation (5) shown below:

(5) $$T_{HEV}=T_{ref}^{M_{1}}+T_{ref}^{M_{2}}+\cdots +T_{ref}^{M_{n}}$$

Equation (5) can be rewritten as follow:

(6) $$T_{HEV}=r_{M_{1}}T_{HEV}+r_{M2}T_{HEV}+\cdots +r_{M_{n}}T_{HEV}$$

where *r_Mi_* is the motor torque contribution and it represents the ratio between the reference operating torque developed by the *i*th traction motor and the overall HEV torque required for traction, as expressed in Equation (7). It is worth noting that all the torque contributions of the *n* used motors sum to one, as highlighted in Equation (8):

(7) $$r_{M_{i}}=\frac{T_{ref}^{M_{i}}}{T_{HEV}}$$

(8) $$\sum_{i=1}^{n}r_{M_{i}}=1$$

With the aim of minimizing ripples occurring during power source switching and during drivetrain commutation from RWD to 4WD, a novel soft transition algorithm is proposed for transient ripple elimination. The different steps are detailed below:

- *Step 1: Determination of the system operating points*

The different motor and power source operating points are defined and set by the designer. The task of defining the system operating points depends on the problem under study. For example, in HEV applications, power source operating points are set via power management algorithms. Equations (9) and (10) show, respectively, that each of the *n* power sources and traction motors shown in Figure 2 can be operated at *n* distinct operating points. It is worth noting that the number of operating points of a given power source or traction motor depends on the problem under study. In this paper, all the power sources and the traction machines are assumed to be the same, for the sake of analysis simplification:

(9) $$\{\begin{array}{c} P_{s1}^{ref}=\left\{r_{1}^{s1}P_{HEV},r_{2}^{s1}P_{HEV},\ldots \;r_{n}^{s1}P_{HEV}\right\}\\ P_{s2}^{ref}=\left\{r_{1}^{s2}P_{HEV},r_{2}^{s2}P_{HEV},\ldots \;r_{n}^{s2}P_{HEV}\right\}\\ \vdots \\ P_{sn}^{ref}=\left\{r_{1}^{sn}P_{HEV},r_{2}^{sn}P_{HEV},\ldots \;r_{n}^{sn}P_{HEV}\right\} \end{array}$$

(10) $$\{\begin{array}{c} T_{M1}^{ref}=\left\{r_{1}^{M1}T_{HEV},r_{2}^{M1}T_{HEV},\ldots \;r_{n}^{M1}T_{HEV}\right\}\\ T_{M2}^{ref}=\left\{r_{1}^{M2}T_{HEV},r_{2}^{M2}T_{HEV},\ldots \;r_{n}^{M2}T_{HEV}\right\}\\ \vdots \\ T_{Mn}^{ref}=\left\{\;r_{1}^{Mn}T_{HEV},\;r_{2}^{Mn}T_{HEV},\ldots \;r_{n}^{Mn}T_{HEV}\right\} \end{array}$$

- *Step 2: Change detection*

For generalization purposes, let *x* denote the HEV variable that will undergo a transition which may be motor torque or power source power, let *Q_i_* denote the set of all its corresponding operating points, and let *k* be a subscript that identifies the power source or the traction machine in which the transition takes place. The reference operating point of a given HEV system variable *x_k_* is expressed as a sum of products of all its possible operating points and their corresponding control signals, as shown in Equation (11):

(11) $$x_{k}^{ref}=\sum_{i=1}^{i=n}C_{i}^{x_{k}}Q_{i}^{x_{k}}\;$$

**Figure 3:**
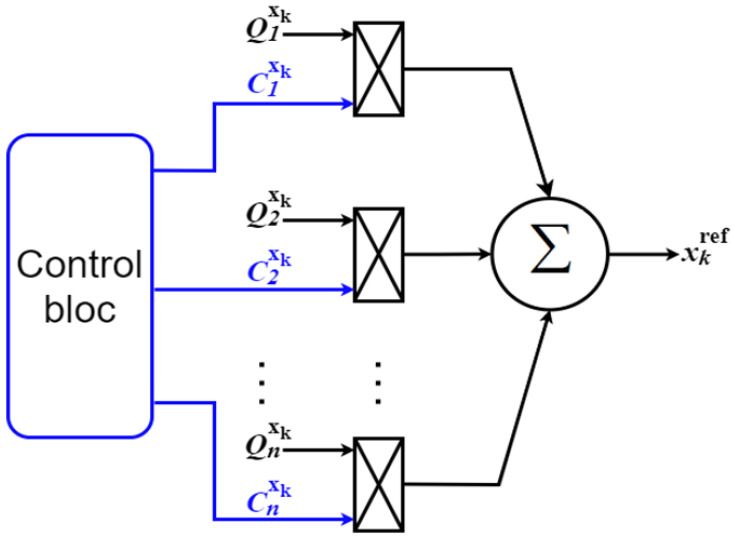
Reference operating point generation.

A graphical interpretation of the above equation is provided in Figure 3. One can note how the different operating points (*Q_i_*) of the system variable *x_k_* are multiplied by their corresponding binary logic control signals (*C_i_*) that are generated by a control block which could be a commutation table, power management algorithm, or any other conditioning circuit depending on the application under study. It is worth noting that only one control signal *C_i_* is high at one time, a change is detected if the state of at least one control signal (*C_i_*) toggles from high to low or vice versa, as illustrated in Equation (12), where *T_s_* is the sampling time period:

(12) $$C_{i}^{x_{k}}(t-T_{s})\neq C_{i}^{x_{k}}(t)$$

- *Step 3: Determination of transition bounds*

When a transition is detected in a given HEV variable *x_k_* at *t* = *t_trig_*, its corresponding operating point (Step one) before transition occurs is stored and set as the lower bound, as shown in Equation (13):

(13) $${\left(x_{k}^{ref}\right)}_{old}=\sum_{i=1}^{i=n}C_{i}^{x_{k}}\left(t_{trig}-T_{s}\right)Q_{i}^{x_{k}}\left(t_{trig}-T_{s}\right)$$

The operating point $Q_{i}^{x_{k}}$ is just a percentage of the HEV variable *x_k_*, as stated in Equation (14):

(14) $$Q_{i}^{x_{k}}=r_{i}^{x_{k}}x_{i}^{x_{k}}$$

Substituting Equation (14) in Equation (13) yields Equation (15):

(15) $${\left(x_{k}^{ref}\right)}_{old}=\sum_{i=1}^{i=n}C_{i}^{x_{k}}\left(t_{trig}-T_{s}\right)r_{i}^{x_{k}}\left(t_{trig}-T_{s}\right)x_{i}^{x_{k}}\left(t_{trig}-T_{s}\right)$$

Using the fact that only one control signal ($C_{i}^{x_{k}}$) is high at one time, and assuming that it is in the *p^th^* position, Equation (15) can be simplified further, and the result is given by Equation (16):

(16) $${\left(x_{k}^{ref}\right)}_{old}=r_{p}^{x_{k}}\left(t_{trig}-T_{s}\right)\;x_{p}^{x_{k}}\left(t_{trig}-T_{s}\right)$$

Following the same approach, the upper bound or the new operating point value of a given variable *x_k_* is given in Equation (17) where it is assumed that the only high control signal *C_i_* is in the *q^th^* position:

(17) $${\left(x_{k}^{ref}\right)}_{new}=r_{q}^{x_{k}}\left(t_{trig}\right)\;x_{q}^{x_{k}}\left(t_{trig}\right)$$

- *Step 4: Determination of transition periods*

The transitions that occur in a given HEV system variable (*x_k_*) are classified into three sets: fast transition $\left(T_{1}^{x_{k}}\right)$, medium transition ($T_{2}^{x_{k}}$), and slow transition $\left(T_{3}^{x_{k}}\right)$ as indicated in Equation (18). This is done mainly to respect the dynamics of the HEV power sources and traction machines:

(18) $$T^{x_{k}}=\left\{ \begin{array}{l} T_{1}^{x_{k}}\;when\;\;0\leq \left|Q_{j}^{x_{k}}-Q_{i}^{x_{k}}\right|\leq B_{1}^{x_{k}}\\ T_{2}^{x_{k}}\;when\;B_{1}^{x_{k}}<\left|Q_{j}^{x_{k}}-Q_{i}^{x_{k}}\right|\leq B_{2}^{x_{k}}\\ T_{3}^{x_{k}}\;when\;B_{2}^{x_{k}}<\left|Q_{j}^{x_{k}}-Q_{i}^{x_{k}}\right|\leq B_{3}^{x_{k}}\\ such\;\;that\;:B_{1}^{x_{k}}<B_{2}^{x_{k}}<B_{3}^{x_{k}} \end{array}\right.$$

- *Step 5: Reference generation*

The proposed switching strategy does not apply the new operating point just after transition detection. Instead, the transition from the old to the new operating point is performed within a defined period using a defined transition function. In this study, two transition functions are proposed. The first function is based on the use of a stair function which is intentionally chosen to split the large ripples and fluctuations noticed during abrupt classical transitions into a series of small and allowable peaks. Another transition function that relies on the use of a linear transition function is used to join the old and the new operating points of a given system variable *x_k_*. When the stair-based transition function (SBT) is used, the points mentioned below are followed to generate a new operating point of a given HEV system variable during [*t_trig_*, *t_trig_* + *T*]. The transition period $T^{x_{k}}$ defined in Step 3 is subdivided into *m_k_* sub-transient periods $\delta^{x_{k}}$, as given in Equation (19):

(19) $$T^{x_{k}}=m_{k}\delta^{x_{k}}$$

After each sub-transient period, the old reference (*x_k_*)*_old_* is increased or decreased by the step given by Equation (20):

(20) $$\Delta Q^{x_{k}}=\left(\frac{Q_{n}^{x_{k}}-Q_{p}^{x_{k}}}{m_{k}}\right)$$

where $Q_{n}^{x_{k}}$ and $Q_{p}^{x_{k}}$ are, respectively, the new and the previous operating points of a given HEV variable *x_k_*.

The targeted operating point is generated using Equation (21), where *i* is an integer ranging from 1 to *m_k_*:

(21) $${\left[x_{k}^{ref}\left(t_{trig}+i\delta^{x_{k}}\right)\right]}_{new}={\left(x_{k}^{ref}\right)}_{old}+i\Delta Q^{x_{k}}$$

**Figure 4:**
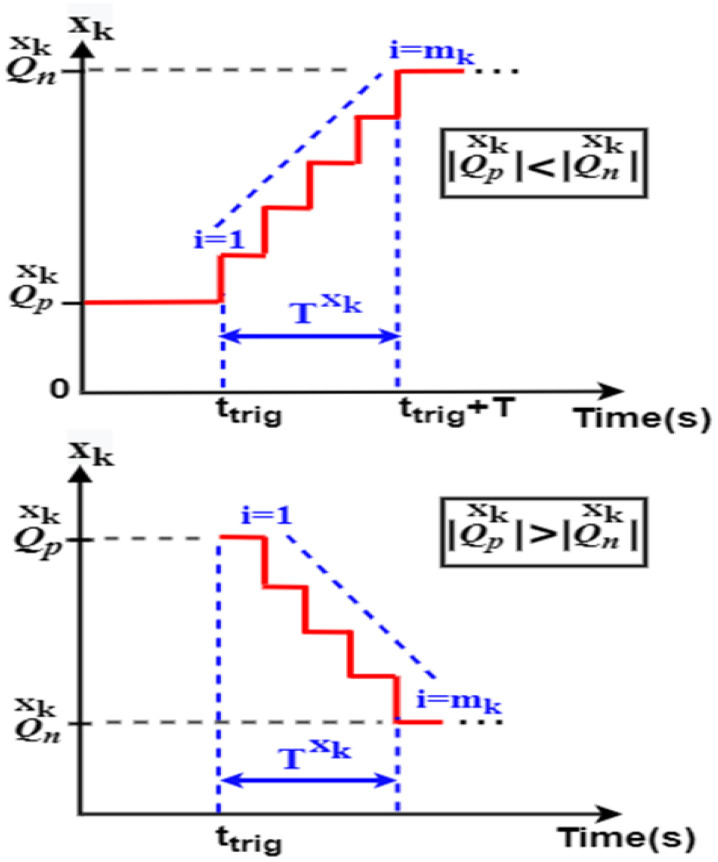
Stair-based transition function.

The first term of the above equation represents the old reference value of the system variable *x_k_*. The second term represents the increase after each sub-transient period. As *i* tends to *m_k_*, Equation (21) converges to the new or targeted operating point. Figure 4 shown below explains graphically how the SBT switches from an old operating point to reach its targeted value.

**Figure 5:**
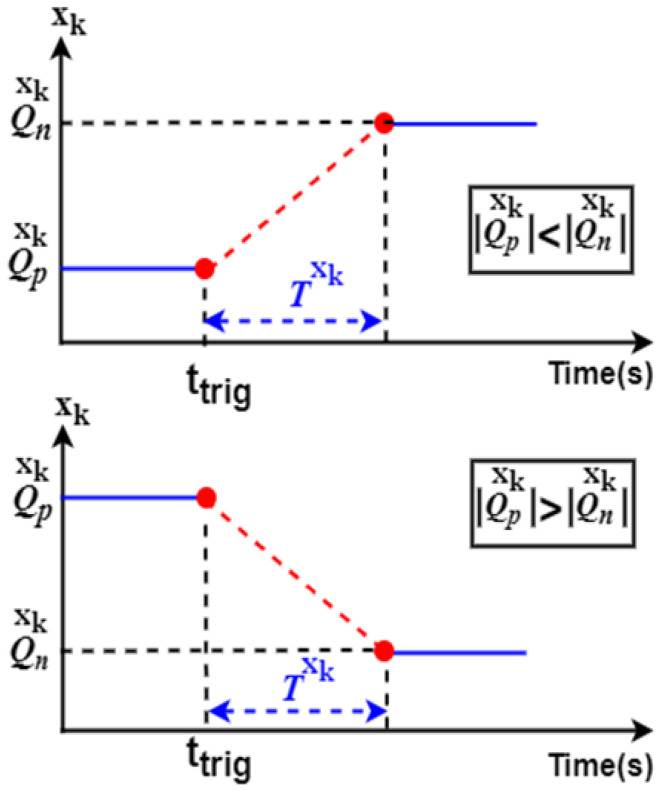
Linear-based transition function.

The linear transition function given by Equation (22) is proposed to join the old and the new operating points of a system variable *x_k_*. When the transition is detected, *Q_p_* will reach its targeted value *Q_n_*, as shown in the equation below (a graphical interpretation is shown in Figure 5):

(22) $$x_{k}^{ref}=\left\{ \begin{array}{l} \left(\frac{Q_{n}^{x_{k}}-Q_{p}^{x_{k}}}{T^{x_{k}}}\right)\left(t-t_{trig}\right)+Q_{p}^{x_{k}}\;;t\in \left[t_{trig},t_{trig}+T\right]\\ Q_{n}^{x_{k}}\;;\;t>t_{trig}+T^{x_{k}} \end{array}\right.$$

**Figure 6:**
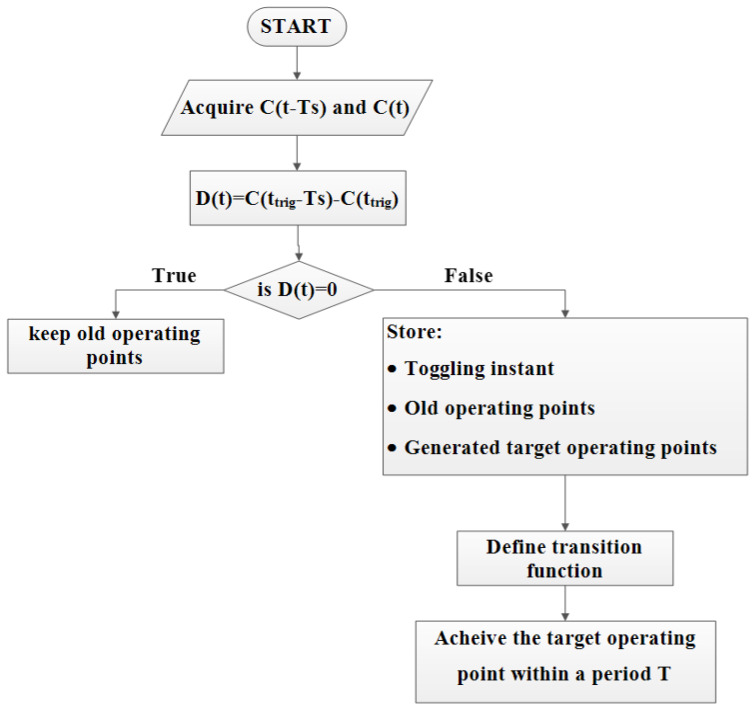
STA flowchart.

The flowchart shown in Figure 6 summarizes the basic or major steps of the STA. It can be deduce that, if no change is detected in the control signal of a given HEV system variable, the STA will not be initiated and the old operating point of a given system variable will be kept. In the case of change detection, the old system operating point will reach its value following the transition model set by the designer.

## 3. Power Sources Modeling

FCs are used extensively in the field of electrical engineering, but, since they deliver power after chemical reactions, they exhibit important delays toward fast load variations. The purifier which is responsible for transforming fuel to pure hydrogen, causes a significant time delay of several seconds. It is worth noting that the purifier’s dynamics are estimated to be of the first order, as highlighted below.

**Figure 7:**
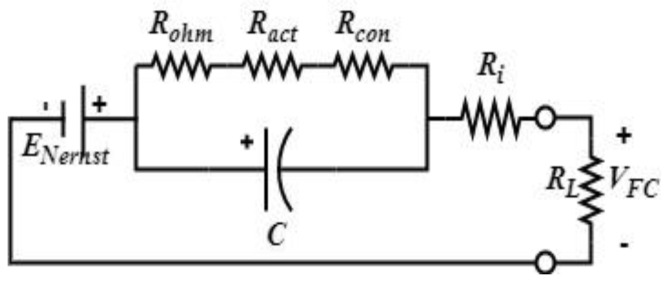
Used FC model.

Figure 7 shows the FC model used in this study. The output voltage *V_FC,CELL_* and the total FC voltage are given by Equations (23) and (24), respectively:

(23) $$V_{FC}=E_{Nernst}-U_{act}-U_{con}-U_{ohm}-R_{L}i(t)$$

(24) $$V_{FC,TOT}=N_{s}V_{FC}$$

where *N_s_* is the number of cells in series, *R_L_* is the load resistance, *U_con_* is the concentration polarization due to the variations in the gradient concentrations at the FC anode and cathode, *U_ohm_* is the ohmic polarization that represents the voltage drop caused by specific FC conductivity, and *U_act_* is the activation polarization due to the slowness of chemical reactions at the two electrodes.

From the above circuit, one can notice that under no load connected to it, the FC has a time constant given by Equation (3), and when it is loaded, its time constant is expressed using Equation (4). The two equations below highlight the slowness of the FC and show that it takes considerable time to respond to transient peak demands. The authors of [27,28] estimated FC slowness to be 2.2$\tau_{FC}^{NL}$ *s* and [3,4] estimated it to be 3$\tau_{FC}^{NL}$ *s*. A FC’s low transient response has to be taken into consideration when it is coupled with other power sources such as batteries and supercapacitors.

(25) $$\tau_{FC}^{NL}=\left(R_{act}+R_{con}+R_{ohm}\right)C$$

(26) $$\tau_{FC}^{FL}=\left[\frac{\left(R_{act}+R_{con}+R_{ohm}\right)(R_{i}+R_{L})}{\left(R_{act}+R_{con}+R_{ohm}+R_{i}+R_{L}\right)}\right]C$$

**Figure 8:**
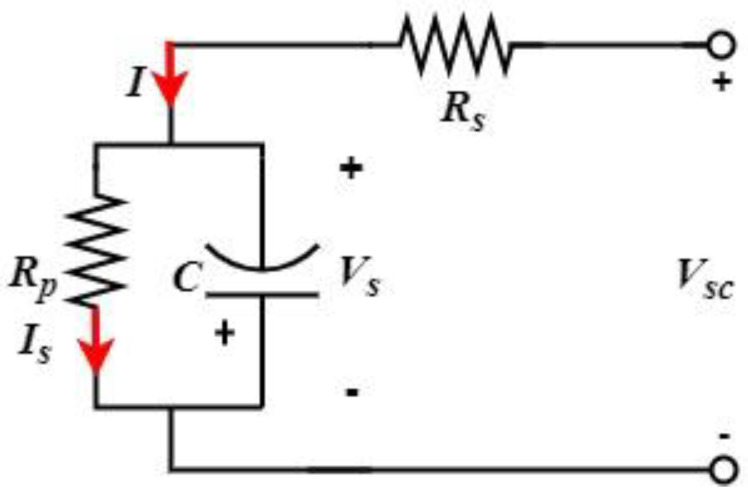
Used SC model.

Supercapacitors are electrical energy storage devices which can be rapidly recharged and can deliver a large amount of power. These storage devices are mainly used in hybrid electric vehicles during accelerations. In the automotive field, they cannot yet compete with Li-ion batteries in terms of energy density, but their capacity continues to be enhanced each year. The equivalent circuit of a supercapacitor is shown in Figure 8 shown below:

The supercapacitor charging and discharging equations are described using Equations (27) and (28) shown below, where *R_P_* is the resistance of the SC parallel branch, *R_S_* is the equivalent series resistance, and *I_S_* is the current that passes through the *R_S_*. The voltage across capacitor C is presented using Equation (29) shown below:

(27) $$\frac{V_{SC}}{V_{S}}=\left[1-e^{-\left(\frac{t}{R_{S}+R_{P}}\right)}\right]$$

(28) $$\frac{V_{SC}}{V_{S}}=e^{-\left(\frac{t}{R_{S}+R_{P}}\right)}$$

(29) $$V_{S}=R_{P}I_{S}$$

## 4. HEV System Based STA

**Figure 9:**
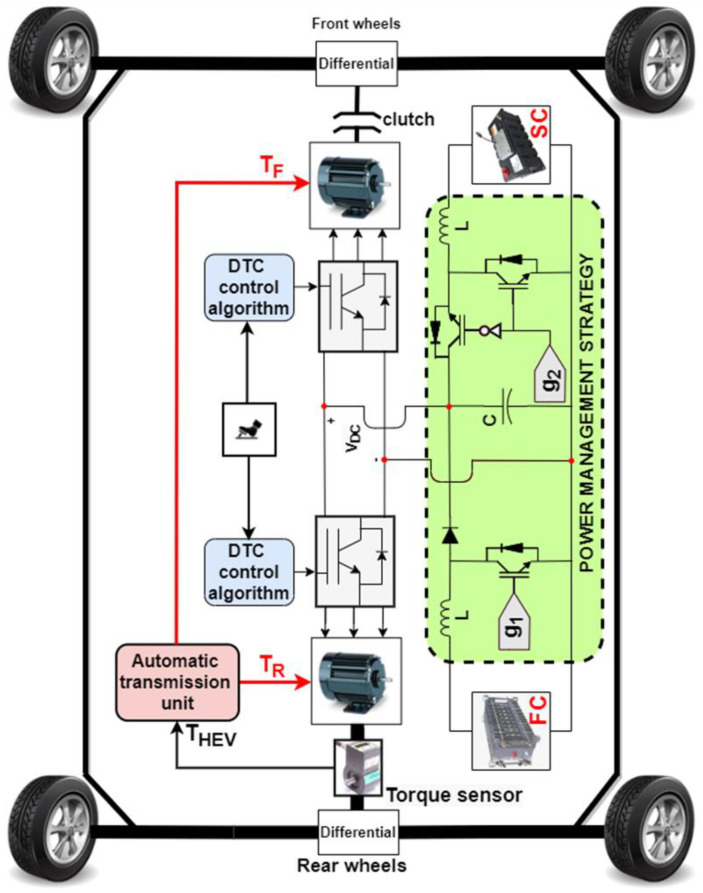
Overview of the FC-SC HEV.

**Figure 10:**
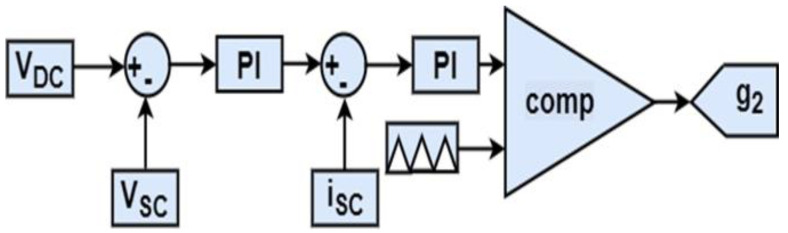
SC control loop.

**Figure 11:**
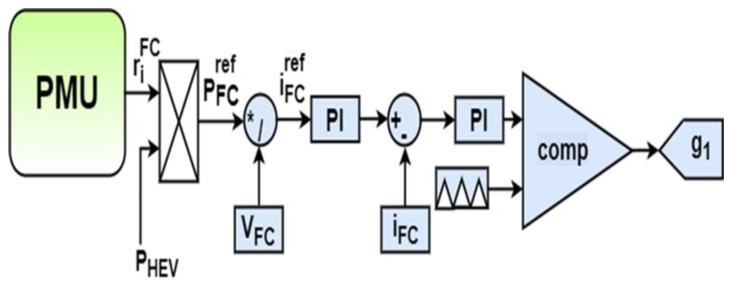
FC control loop.

The proposed soft transition algorithm is applied, as shown in Figure 9. It can be seen that the HEV architecture consists of two PMSMs controlled using direct torque control (DTC). This drivetrain arrangement makes it possible for the HEV to operate either in rear-wheel mode (RWD) or in four-wheel drive mode (4WD). The driving mode depends on the value of load torque applied on the vehicle. If the torque is below a specified threshold value (*T_TH_*), the HEV will run in RWD. In this situation, the shaft of the front PMSM will be disconnected from the front wheels and only the rear PMSM ensures HEV traction and handles all the load torque applied on the vehicle. As soon as the torque applied on the HEV exceeds *T_TH_*, the front PMSM shaft will be automatically connected to the front wheels and the HEV will run in 4WD. In this case, each PMSM ensures half of the load torque *T_HEV_*. Commutations from RWD to 4WD, and vice versa, imply abrupt connection and disconnection of the motor shaft from the vehicle’s front wheels. This will result in significant torque ripples which reduce riding comfort. The SC and FC are, respectively, connected to the DC bus via bidirectional and unidirectional DC-DC converters. The gate signals, g_1_ and g_2_, shown in the green-dashed area, are generated from the two control loops shown in Figure 10 and Figure 11, respectively. It can be noted that the SC is used to regulate the DC bus voltage at its reference. The power management unit (PMU), shown in green in Figure 9, outputs a factor $r_{i}^{FC}$ which represents the percentage of the total power that the FC will deliver. This will avoid deep FC discharge and enhance HEV autonomy.

The unwanted torque ripples that occur during RWD/4WD transitions and the high power peaks that occur when switching between power sources will significantly reduce driving comfort. Hence, the proposed STA, presented in the previous section, will be applied for comfort enhancement and minimization of the ripples that occur during the different HEV transitions.

**Figure 12:**
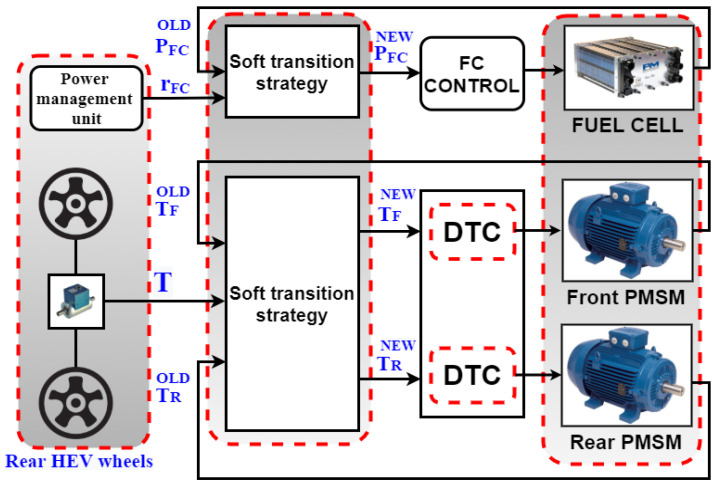
Proposed HEV based STA scheme.

In order to link between the FC- SC HEV system presented in Figure 9 and the STA theory described in Section 2, Figure 12 is presented to show how the STA is adapted for use with an HEV and how the vehicle is seen by the STA.

By analogy between Figure 3 and Figure 12, it can be deduced that the STA block receives three HEV parameters and their corresponding control signals. As stated in Step 1 of Section 2.2, the possible operating points of each HEV system variable are defined, as described below:

- FC power (*P_FC_*) is an HEV parameter controlled using the STA. As shown in Figure 10, the number of FC operating points is determined by the power management unit (PMU) which will output the FC power contribution factor *r_RF_* that will make the FC operate at one of the five distinct operating points shown in the equation below:
  (30) $$P_{FC}^{ref}=\left\{r_{1}^{FC}P_{HEV},r_{2}^{FC}P_{HEV},r_{3}^{FC}P_{HEV},r_{4}^{FC}P_{HEV},r_{5}^{FC}P_{HEV}\right\}$$
- For the front motor torque (*T_FM_*) parameter, when the torque applied on the HEV exceeds a specified threshold value *T_TH_*, the front motor will be ON and will handle half of the torque applied on the vehicle. Otherwise, it will be OFF. Hence, the set of front PMSM operating points are given in Equation (31) shown below:
  (31) $$T_{FM}^{ref}=\left\{r_{1}^{FM}T_{HEV},r_{2}^{FM}T_{HEV}\right\}$$
- For the Rear motor torque (*T_RM_*) parameter, the rear PMSM will ensure alone HEV traction when the load torque applied on the HEV is less than a specified threshold torque value *T_TH_*. As soon as *T_HEV_* exceeds the threshold value, the rear traction machine while ensure only half of the torque needed for HEV traction. Hence, the set of rear PMSM operating points consists of two values, as shown in Equation (32):
  (32) $$T_{RM}^{ref}=\left\{r_{1}^{RM}T_{HEV},r_{2}^{RM}T_{HEV}\right\}$$

The corresponding numerical values of the FC power contribution factor previously mentioned in Equation (23) are given in Table 1 and the torque contribution factors of the front and rear PMSMs are highlighted in Table 2. For example, when $r_{3}^{FC}=50\%$, this means that the FC will deliver half of the required power for traction and the other half will be ensured by the SC and, in the case where $r_{2}^{RM}=100\%$, this means that all the torque will be handled by the rear traction machine.

When the HEV torque exceeds *T_TH_*, the HEV will toggle from RWD to 4DW and the front PMSM will be assigned half of the torque applied on the vehicle. Performing this with classical binary switching will cause significant torque ripples and will reduce the driving comfort, which is demonstrated later. Using the STA, the front motor smoothly reaches its reference using Equation (33) when the SBT is used as a transition function. Equation (34) can be used to ensure smooth torque transition in the case where the LBT is used as a transition function.

(33) $$T_{FM}^{REF}\left(t_{trig}+i\delta^{FM}\right)=T_{FM}^{old}+i\left(\frac{Q_{n}^{\;new}-Q_{p}^{\;old}}{m_{FM}}\right)T_{HEV}$$

(34) $$T_{FM}^{REF}\left(t\right)=\left(\frac{Q_{FM}^{\;new}-Q{\;}_{FM}^{\;old}}{T^{T_{FM}}}\right)\left(t-t_{trig}\right)+T{\;}_{FM}^{\;old}$$

In the two above equations, $T_{FM}^{old}$ is a constant value representing the front PMSM torque reference value, one sample time before the transition from RWD to 4WD, as already stated in Equation (16), $\delta^{FM}$ is the front motor sub-transient period, $T^{T_{FM}}$ is the motor switching period, and *i* is an integer ranging from 1 to *m_FM_*. The STA is also applied to the rear PMSM. Any increase or decrease in the rear PMSM torque is indirectly controlled by the STA, as can be noted from Equation (35):

(35) $$T_{RM}^{REF}=T_{L}-T_{FM}^{REF}\left(t_{trig}+i\delta^{FM}\right)$$

Equation (36) highlights the transition function used when the FC toggles between two distinct operating points using the SBT function, and Equation (37) represents the linear transition function that ensures FC power toggling from one operating point to another. In Equation (36), *j* is an integer ranging from 1 to *m_FC_*:

(36) $$P_{FC}^{REF}\left(t_{trig}+j\delta^{FC}\right)=P_{FC}^{\;old}+j\left(\frac{Q_{FC}^{\;new}-Q{\;}_{FC}^{\;old}}{m_{FC}}\right)P_{HEV}$$

(37) $$P_{FC}^{REF}\left(t\right)=P_{HEV}\left(\frac{Q_{FC}^{\;new}-Q{\;}_{FC}^{\;old}}{T^{P_{FC}}}\right)\left(t-t_{trig}\right)+P_{FC}^{\;old}$$

The SC power is calculated using Equation (38). The previous equation shows that the SC power is indirectly controlled using the STA through control of the FC power:

(38) $$P_{SC}^{REF}=P_{HEV}-P_{FC}^{REF}$$

## 5. Simulations and Results

The used parameter simulation and HEV parameters are shown in Table 3 and Table 4, respectively; all simulations were performed using MATLAB-Simulink 2015b with a fixed step solver.

**Figure 13:**
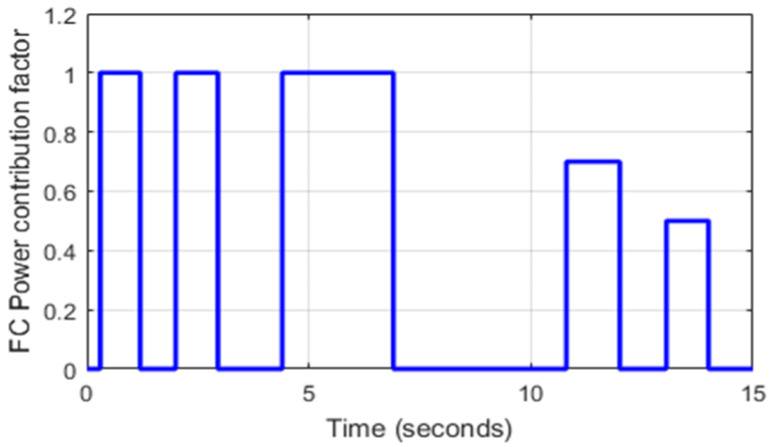
FC power contribution factor.

The PMU unit, shown in Figure 13, outputs different desired FC power contribution factors ($r^{FC}$), as shown in the control loop in Figure 11. Note that $r^{FC}$ is variable and varies from 0% to 100%, as already stated in Table 1. For example, when $r^{FC}=0.7$, it means that the fuel cell ensures 70% of the required power for traction.

**Figure 14:**
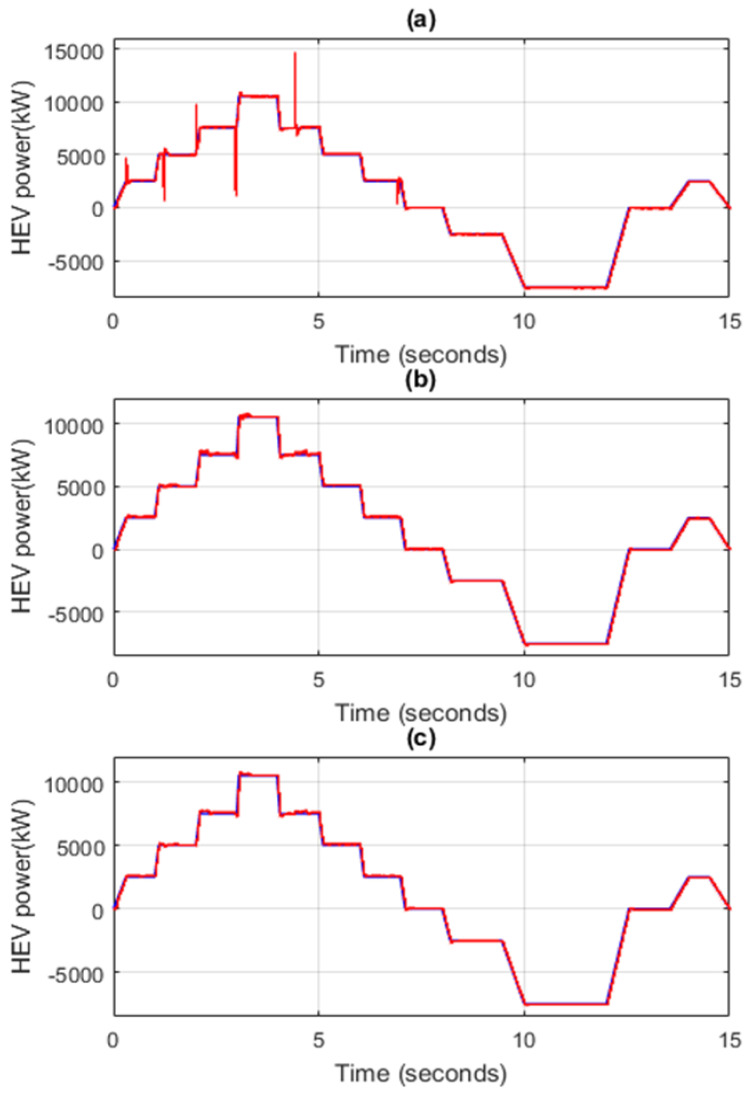
HEV power using different transition techniques. (**a**) Classical abrupt switching (**b**) SBT switching (**c**) LBT switching.

Figure 14 shows the HEV power, when switching between power sources is performed using different transition strategies. Figure 14a shows the obtained HEV power, when switching between SC and FC is performed using *classical abrupt switching*. Figure 14b and Figure 14c represent the resulting HEV power, when the switching between vehicle power sources is performed using the SBT and LBT techniques, respectively. Note that the SBT and LBT techniques result in suppression of the large ripples that occur during power source switchings.

**Figure 15:**
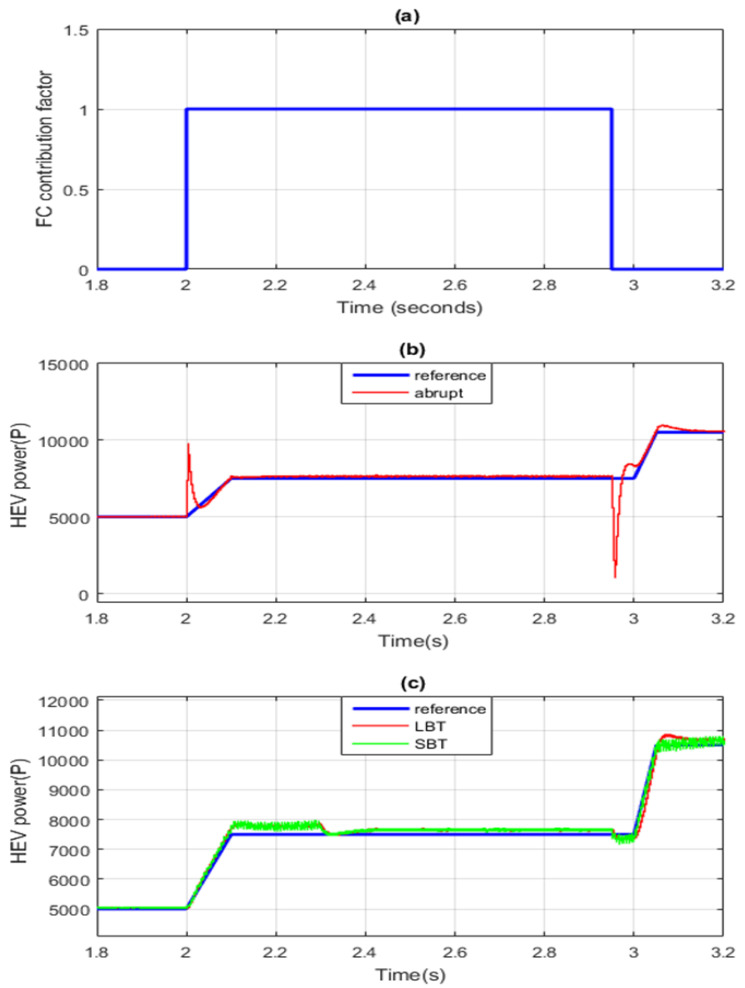
Zoom of HEV power. (**a**) Abrupt switching (**b**) SBT switching (**c**) LBT switching.

Figure 15a represents a zoom of the FC power contribution factor shown in Figure 13. It can be seen that the FC alone ensures all HEV power required for traction during [2 s, 2.95 s]. Figure 15b shows a zoom of the HEV power when classical abrupt switching is used, and Figure 15c shows a zoom of vehicle power when the commutations between SC and FC are performed using the SBT and LBT transition functions. Note that the abrupt connection and disconnection of FC at *t* = 2 s and *t* = 2.95 s caused a very large and harmful power peak of almost 5 kW, whereas the FC connections and disconnections through the use of SBT and LBT transition functions suppressed the large ripples during power source switching.

**Figure 16:**
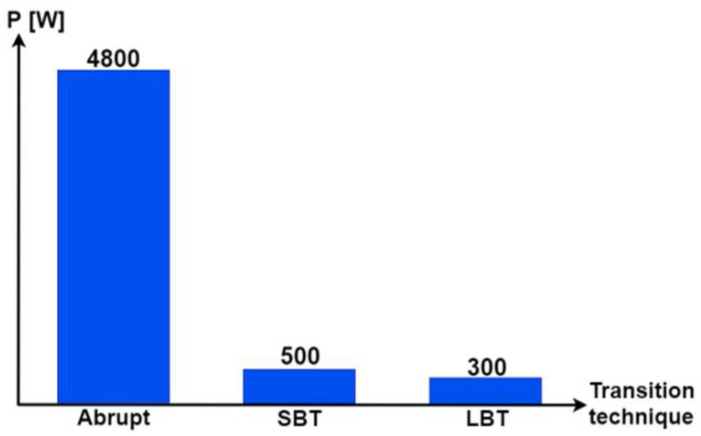
Ripples with different transition techniques.

Figure 16 confirms that the STA minimizes losses during the power source transitions. It can be concluded that the power peak ripples are minimized almost ten times using STA-based switching. LBT resulted in minimum transient ripples.

**Figure 17:**
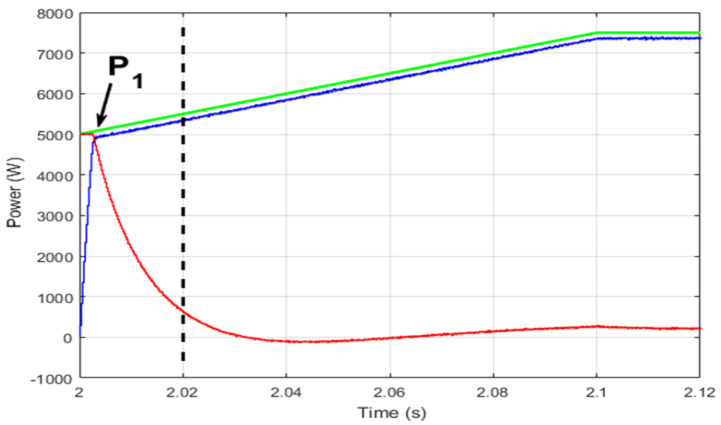
Classical abrupt power source switching.

To investigate the cause of the large power fluctuations, a zoom of the instant during which the FC is turned on and the SC is turned off at *t* = 2 s is studied. From Figure 17, one can observe that just after the FC is turned on, its power reaches the required HEV power, while the SC has not yet discharged. This fact violates the power conservation equation given by Equation (36), and this is seen explicitly in the area to the left of the dark-dashed line in Figure 17; the sum of power produced by SC and FC is almost twice the required power for the traction highlighted in green. This phenomenon reduces HEV comfort.

**Figure 18:**
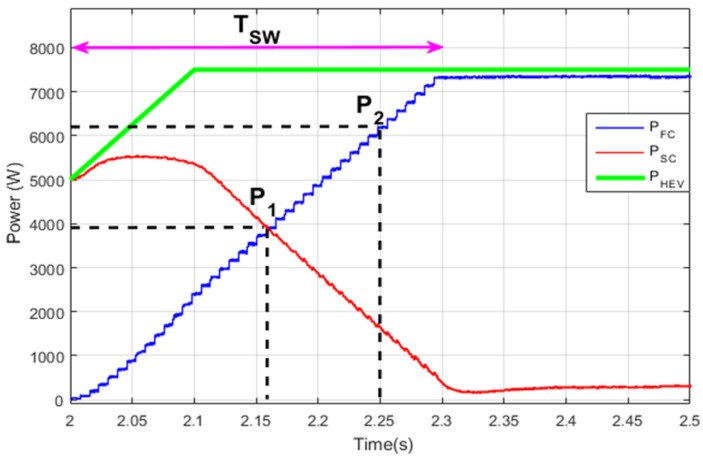
Stair-based power source switching.

Figure 18 shows the instant at which the FC is turned on to provide, alone, all the traction power using the STA-based stair transition function. One can see how the transition is split into small allowable sub-transitions performed in T_SW_. The transition is performed smoothly, and the power conservation equation, given by Equation (36), is respected at every point in [2, 2 + T_SW_]. Two points P_1_ and P_2_ are provided to highlight explicitly how the SC and FC power sum to the HEV required power at every point in the transition period.

**Figure 19:**
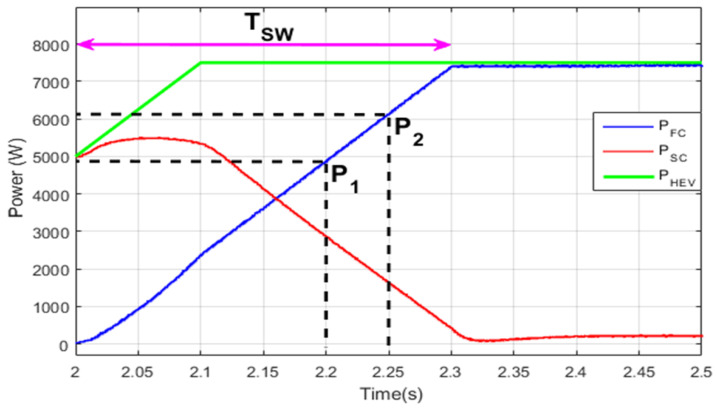
Linear-based power source switching.

Figure 19 represents the transition period where the FC is turned on using the LBT transition function. This technique also results in suppression of the large power ripples when the FC is turned on and the HEV power sources are turned off. Again, it can be seen that the power conservation equation given in Equation (31) is respected every instant, such as P1 and P2.

**Figure 20:**
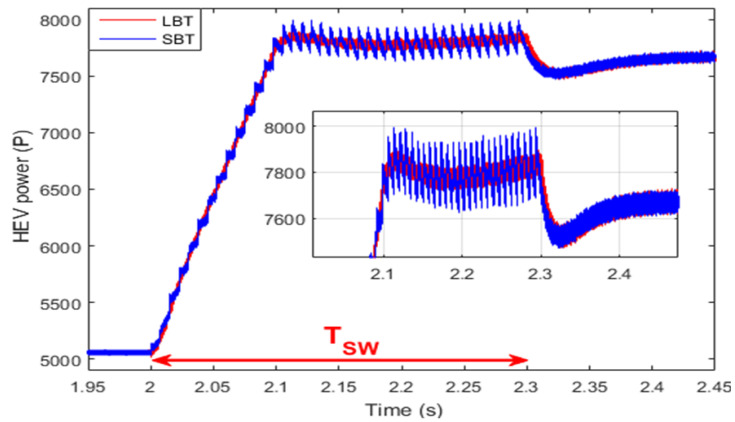
Control system-based STA.

**Figure 21:**
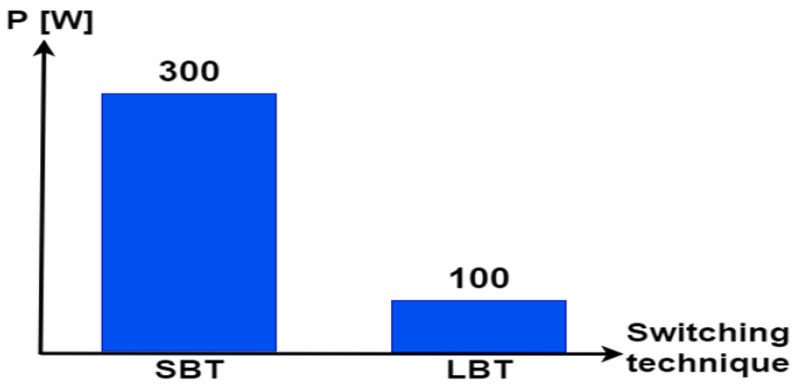
HEV power ripples SBT vs. LBT.

So far, applications of the LBT and SBT transition functions seem to have the same effect on the HEV system, which is the suppression of the harmful ripples noticed during the commutation between HEV power sources. In order to investigate the difference between these two transition techniques, the sum of the SC and FC powers, previously shown in Figure 18 and Figure 19, is presented in Figure 20 during the time interval [2, 2 + T_SW_]. Notice that the hysteresis power band obtained using the LBT and SBT functions are both tolerable, but the LBT is better in terms of ripple minimization since it results in a hysteresis band which is almost three times smaller than the one obtained using the SBT function, as highlighted in Figure 21. The reason why the SBT function results in a larger hysteresis torque band is because the SBT function is no more than an abrupt classical transition that is split into *m_FM_* small sub-transitions which, in turn, cause relatively high ripples as compared with the LBT.

**Figure 22:**
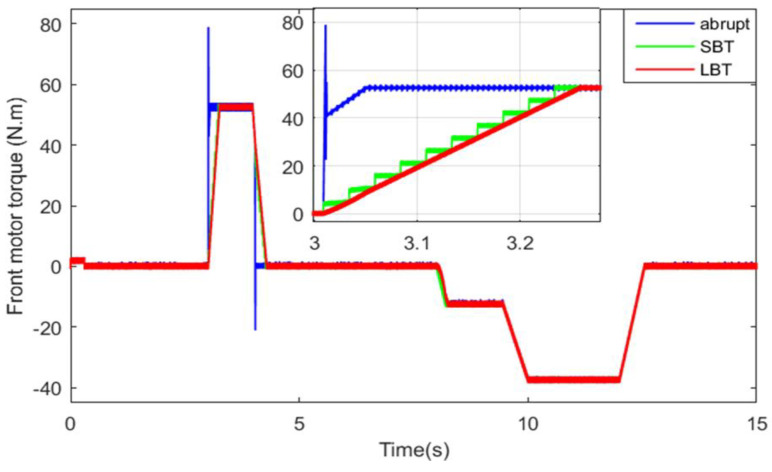
Front motor torque.

The electromagnetic torque developed by the front traction machine is shown in Figure 22. It can be seen that the front machine remains “*off*” or disconnected from the front wheels until the instant during which the load torque applied on the HEV reaches and exceeds the set threshold value *T_TH_* at *t* = 3.02 s. In this case, the front machine *torque contribution factor* will toggle from *r_FM_* = 0 to *r_FM_* = 50%, the front machine will be connected to the front wheels, and it will ensure half of the torque applied on the vehicle. It is worth noting that the HEV drivetrain architecture will toggle from RWD to 4WD as soon as the front machine is triggered.

**Figure 23:**
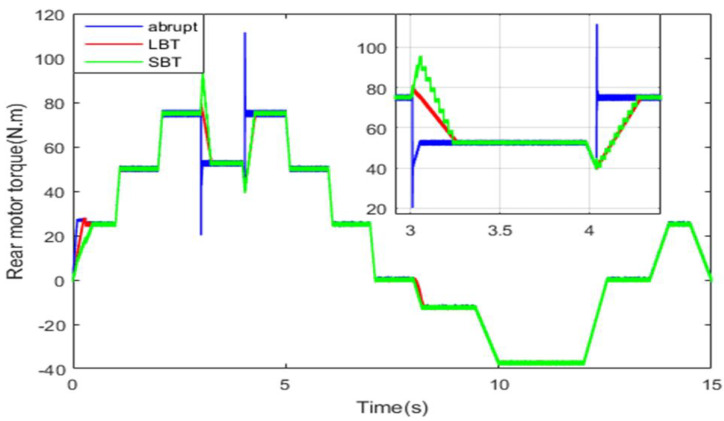
Rear motor torque.

The torque developed by the rear machine is shown in Figure 23. This machine alone ensures the HEV traction when the load torque value is less than the threshold *T_TH_*. As soon as the torque value exceeds *T_TH_*, it can handle half of the torque applied on the HEV and the remaining torque is handled by the front machine.

One can note from Figure 22 and Figure 23 that at *t* = 3.02 s, the load torque applied on the HEV exceeds the specified threshold, and hence the front PMSM is connected to HEV wheels. At *t* = 4.05 s, the load torque decreases below the threshold torque value, and hence the front PMSM is *turned off* and disconnected from the front wheels. Note that when the front PMSM is *turned on*/*off* using classical abrupt switching, high torque ripples are noticed, as can be seen in the zooms of Figure 22 and Figure 23. However, when the motor switching is performed using the LBT and SBT transition functions, no ripples are present and the RWD to 4WD toggling is smoothly performed within the specified time period. Table 5 confirms that the SBT and LBT functions have suppressed the transient torque ripples noticed during drivetrain transitions. During regenerative braking periods, both PMSMs are used to restitute the maximum amount of energy.

**Figure 24:**
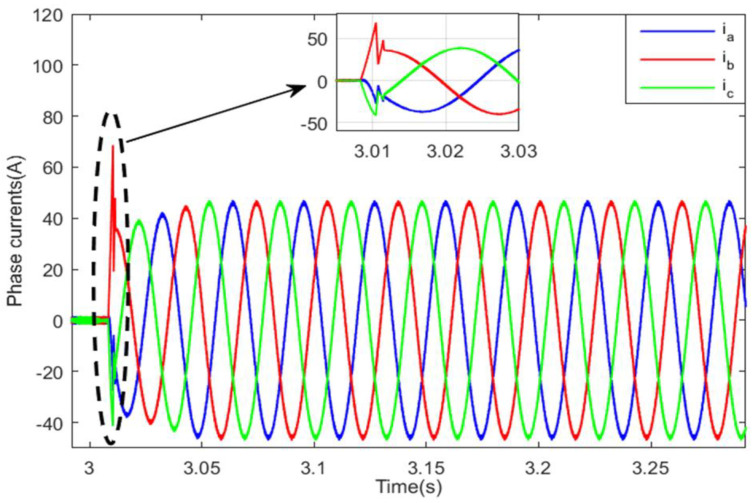
Resulted PMSM currents with abrupt switching.

**Figure 25:**
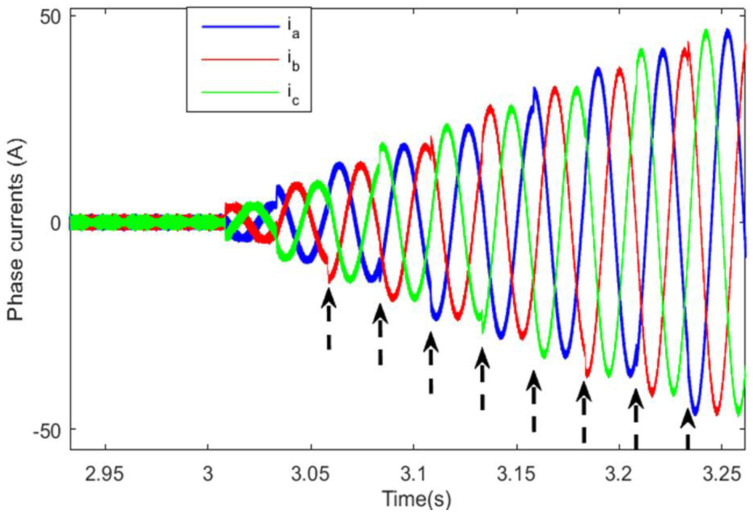
Front PMSM currents with the SBT.

**Figure 26:**
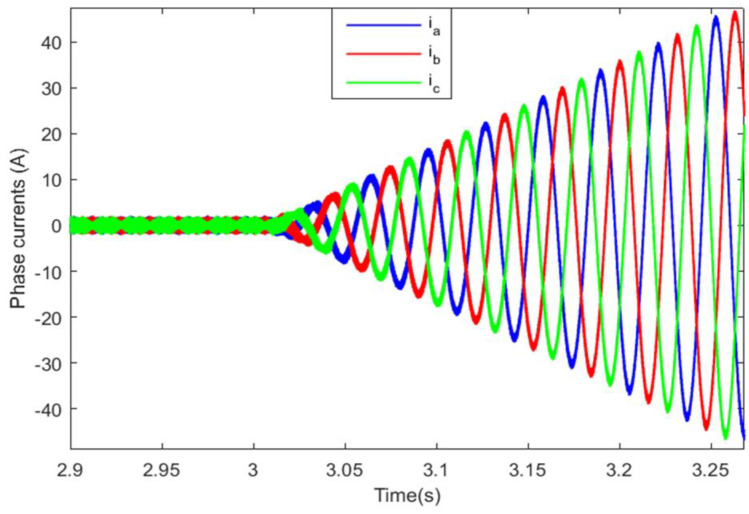
Front PMSM currents with the LBT function.

The abrupt torque variation also resulted in significant current ripples in the front machine, as can be seen in Figure 24. The application of the SBT function for motor switching has enhanced the front PMSM current shape, but there is still cyclic tolerable current deformation that occurs each sub-transient period $\delta^{T_{FM}}$, as shown in Figure 25. Figure 26 shows the front PMSM current, when motor switching is performed using the LBT function, the currents are smooth and gradually increasing, and this increases the riding comfort.

**Figure 27:**
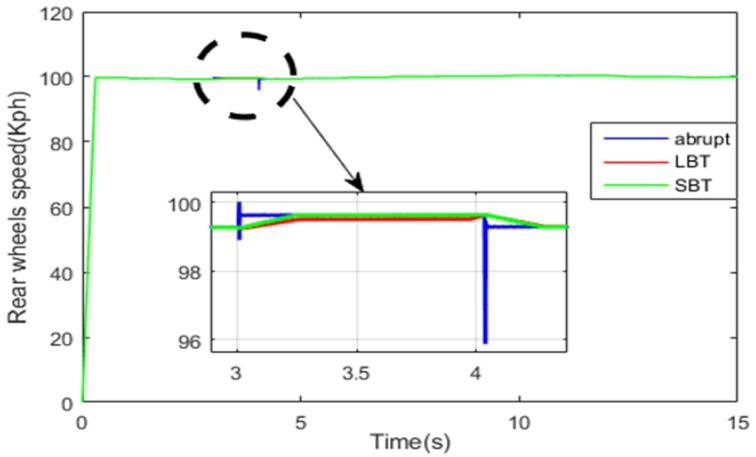
Rear-wheel speed.

**Figure 28:**
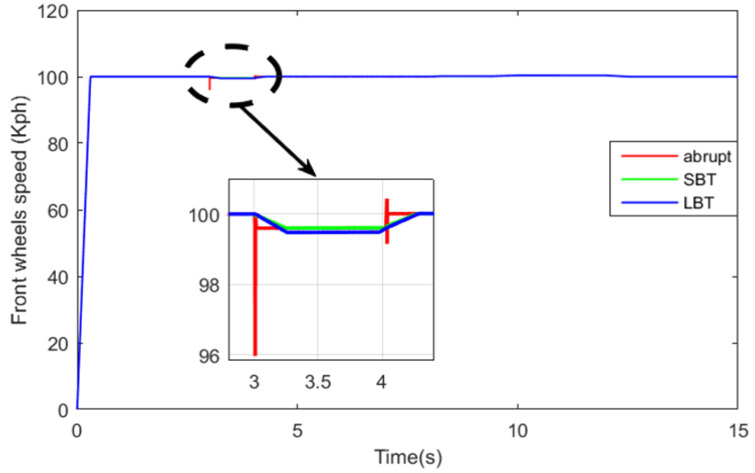
Front-wheel speed.

Figure 27 and Figure 28 show the front- and rear-wheel speeds. All vehicle wheels follow their reference speed. The HEV speed is disturbed when its traction mode toggles from RWD to 4WD during the time interval [3.02 s, 40.5 s]. It can be seen from the previous two figures that the use of the SBT and LBT functions enhance the disturbance rejection of the HEV, and hence increase the comfort of the vehicle.

**Figure 29:**
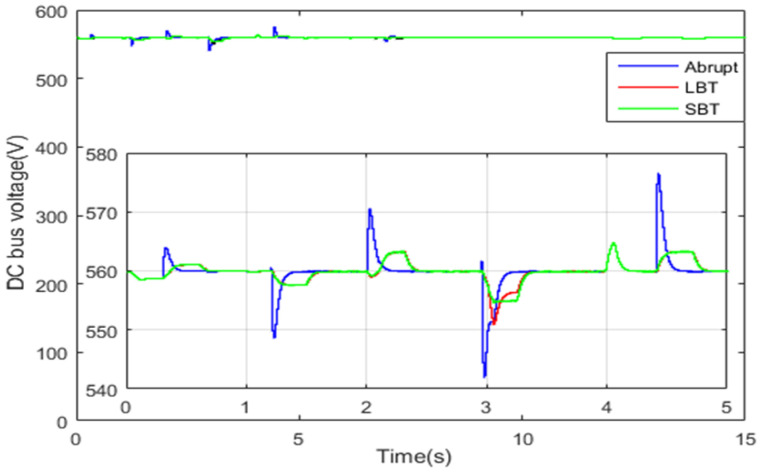
DC bus voltage.

DC bus voltage is shown in Figure 29. The resulting DC bus voltage ripples when the SBT and LBT functions are used for power source switching are very small and lay within a narrow hysteresis band of 5 volts. However, when abrupt classical switching is used for the HEV power source switching, large voltage fluctuations of about 20 volts are noticed. Hence, the STA-based switching results in minimizing DC bus ripples too. One can clearly conclude that the proposed soft transition algorithm has reduced the transient DC bus ripples by 25% as compared with the classical abrupt switching.

## 6. Conclusions

Comfort is an important design factor that precedes vehicle commercialization. In this study, HEV comfort and transient performance were significantly enhanced after the application of the proposed STA. This new proposed technique makes it possible for a designer to model and handle the different transitions that occur in an HEV using transition functions. The proposed LBT and SBT functions showed their superiority as compared with binary classical switching; they solved the problem of high transient peaks that occur during transitions. The transition functions resulted in minimizing the FC and SC power ripples, and this would increase their lifetime. DC bus voltage fluctuations and PMSM current peaks during transitions were also minimized as a result of the application of the proposed algorithm. The proposed soft transition strategy reduced the transient DC bus ripples by 25% as compared with classical abrupt switching. Furthermore, vehicle speed and torque ripples were significantly tightened as a result of application of the proposed transition strategy. For instance, transient torque jerks that reduce driving comfort were reduced by 35 N·m. As a further work, we plan to study the effect of the proposed soft transition algorithm on other engineering systems.

## Figures and Tables

**Table 1:** Different FC power contribution factors.

Factor	$r_{1}^{FC}$	$r_{2}^{FC}$	$r_{3}^{FC}$	$r_{4}^{FC}$	$r_{5}^{FC}$
Value	0%	30%	50%	70%	100%

**Table 2:** Rear and front torque contribution factors.

Factor	$r_{1}^{FM}$	$r_{2}^{FM}$	$r_{1}^{RM}$	$r_{2}^{RM}$
value	0%	50%	50%	100%

**Table 3:** Different simulation parameters.

Symbol	Value
*T_S_*	1 × 10^−6^ s
*T_TH_*	80 N·m
$T^{P_{FC}}$	0.3 s
$T^{T_{FM}}$	0.25 s
*m_FM_*, *m_RM_*	40
*m_FC_*	7.5 ms

**Table 4:** Used HEV parameters.

Parameter	Value
Weight	1200 Kg
Wheel radius	0.32 m
Maximum torque	126 N·m
Frontal area	2.6 m^2^
Air density	1.2 kg/m^3^
Aerodynamic coefficient	0.3

**Table 5:** Torque ripples using all transition functions.

Transition Technique	Maximum Transient Torque Ripples	
	Front Motor	Rear Motor
Abrupt	35 N·m	36.5 N·m
LBT	0	0
SBT	0	0

## Data Availability

Not applicable.
